# Synthesis and structure of a tetra­hedral homoleptic Cu^I^ complex with xylyl isocyanide

**DOI:** 10.1107/S2056989025009867

**Published:** 2025-11-18

**Authors:** A. M. Buddhika Chandima, Stanislav Groysman, Cassandra L. Ward

**Affiliations:** a Department of Chemistry Wayne State University, 5101 Cass Avenue Detroit Michigan 48202 USA; b Lumigen Instrument Center Wayne State University, 5101 Cass Avenue Detroit Michigan 48202 USA; Universidad de la República, Uruguay

**Keywords:** copper(I) isocyanide, Mo/Cu–CO de­hydrogenase, crystal structure, π–π stacking, Hirshfeld surface analysis

## Abstract

Treatment of the Cu^I^ precursor [Cu(NCMe)_4_]PF_6_ with excess (10 equivalents) of relatively bulky xylyl isocyanide formed a tetra­(isocyanide) complex, namely, tetra­kis­(2,6-di­methyl­phenylisocyanide)copper(I) hexa­fluoro­phosphate, [Cu(C_9_H_9_N)_4_]PF_6_ or [Cu(CNX­yl)_4_]PF_6_, in good yield. This is in contrast to the previously reported reactions of Cu^I^ precursors with approximately three equivalents of xylyl isocyanide, which led selectively to the formation of tris­(isocyanide) complexes. The copper atom lies on a twofold axis and P atom on an inversion centre. The complex was characterized by X-ray crystallography, IR spectroscopy, and ^1^H/^13^C {^1^H} NMR spectroscopy. In the crystal structure, each individual [Cu(CNX­yl)_4_]^+^ mol­ecule demonstrates two pairs of coplanar xylyl isocyanide ligands. This arrangement leads to inter­molecular π-stacking inter­actions between nearby complex mol­ecules.

## Abstract 2

Treatment of a tetra­(aceto­nitrile)­copper(I) precursor with excess xylyl isocyanide forms the title tetra­(isocyanide)copper(I) complex, which was characterized structurally and spectroscopically.

## 1. Chemical context

There is a significant inter­est in Cu^I^ complexes in poly(isocyanide) ligands environments. Tris(isocyanide) Cu^I^ complexes have been shown to serve as versatile platforms for catalysis and small-mol­ecule activation (Melekhova *et al.*, 2015 ▸; Ferraro *et al.*, 2021 ▸, 2023 ▸; Kinzhalov *et al.*, 2022 ▸; Kinzhalov *et al.*, 2023 ▸), whereas tetra­(isocyanide) Cu^I^ complexes serve as nodal points in photoactive materials and MOFs, or function as transmetallation reagents (Bartholomew *et al.*, 2022 ▸; Balto *et al.*, 2021 ▸; Claude *et al.*, 2023 ▸; Ruiz & Mateo, 2022 ▸). A survey of the Cambridge Structural Database appears to demonstrate a general trend in which non-bulky aryl isocyanide ligands (lacking ortho substituents) form tetra­kis­(isocyanide) Cu^I^ complexes (Bartholomew *et al.*, 2022 ▸; Balto *et al.*, 2021 ▸; Claude *et al.*, 2023 ▸; Ruiz & Mateo, 2022 ▸; Perrine *et al.*, 2010 ▸), whereas somewhat bulkier *ortho*-disubstituted xylyl (2,6-di­methyl­phen­yl) isocyanide (CNX­yl) forms tris­(isocyanide) Cu^I^ complexes adopting a trigonal-planar or trigonal-monopyramidal geometry (Melekhova *et al.*, 2015 ▸; Ferraro *et al.*, 2021 ▸, 2023 ▸; Kinzhalov *et al.*, 2022 ▸, 2023 ▸). Notably, bulkier CN(2,6-Mes_2_C_6_H_3_) (Mes = mesityl, 2,4,6-Me_3_C_6_H_2_) was shown to demonstrate a preference for tris­(isocyanide) ligation and a trigonal-planar geometry [in Cu(CNAr)_3_(THF)], although a closely related *para*-functionalized CN(2,6-Mes)_2_C_4_H_3_-4-C_6_H_4_CO_2_H was also capable of forming a tetra­(isocyanide) Cu^I^ complex under certain conditions (Balto *et al.*, 2021 ▸; Fox *et al.*, 2008 ▸). We also note that Walton, Edwards, and coworkers reported spectroscopic characterization of a tetra­kis­(xylylisocyanide) Cu^I^ complex; however, these findings were not supported structurally (Bell *et al.*, 1985 ▸). We are pursuing Cu^I^ isocyanide chemistry as part of an investigation into the functional models of Mo–Cu CO de­hydrogenase (Dobbek *et al.*, 2002 ▸; Kaluarachchige Don *et al.*, 2021 ▸, 2023*a* ▸,*b* ▸, 2024 ▸; Hollingsworth *et al.*, 2018 ▸; Chandima *et al.*, 2025 ▸). As part of this project, we became inter­ested in the synthesis of homoleptic Cu^I^ precursors with relatively bulky isocyanide ligand CNXyl (Xyl = 2,6-di­methyl­phen­yl). Herein we demonstrate that the reaction of Cu^I^ precursor with excess CNXyl invariably leads to the formation of [Cu(CNX­yl)_4_]^+^. The structural and spectroscopic characterization of this complex are reported.
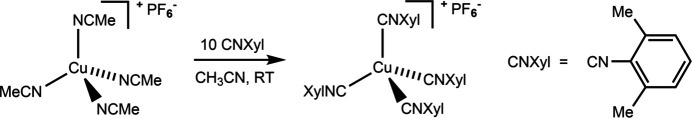


[Cu(CNX­yl)_4_]PF_6_ is formed by the reaction between [Cu(NCMe)_4_]PF_6_ with excess (10 equivalents) of xylyl isocyanide, followed by recrystallization from di­chloro­methane/ether solution. [Cu(CNX­yl)_4_]PF_6_ was characterized by ^1^H and ^13^C {^1^H} NMR spectroscopy, FT-IR spectroscopy, and X-ray crystallography. The spectroscopic data are consistent with the single species in solution. ^1^H NMR demonstrates three resonances in a 1:2:6 ratio: triplet for the single proton in the *para* position of the xylyl ligand, doublet for the two *meta* protons, and a singlet for the two methyl groups. The ^13^C {^1^H} NMR spectrum (CD_2_Cl_2_) demonstrates four aromatic signals, an aliphatic signal, and a signal at 146.47 ppm suggestive of coordinated isocyanide (Ferraro *et al.*, 2021 ▸). IR (ATR) features a signal at 2153 cm^−1^ consistent with the C≡N(Ar) coordinated to a non π-basic Cu^I^ (Ferraro *et al.*, 2021 ▸; Chandima *et al.*, 2025 ▸; Kaluarachchige Don *et al.*, 2021 ▸, 2023*a* ▸,*b* ▸, 2024 ▸; Hollingsworth *et al.*, 2018 ▸); this signal appears at slightly lower frequency compared with the previously reported [Cu(CNX­yl)_3_]^+^ (υ_CN_ = 2170 cm^−1^). No signals consistent with the presence of free isocyanide, or other metal species were observed by NMR or IR.

## 2. Structural commentary

**Figure 1:**
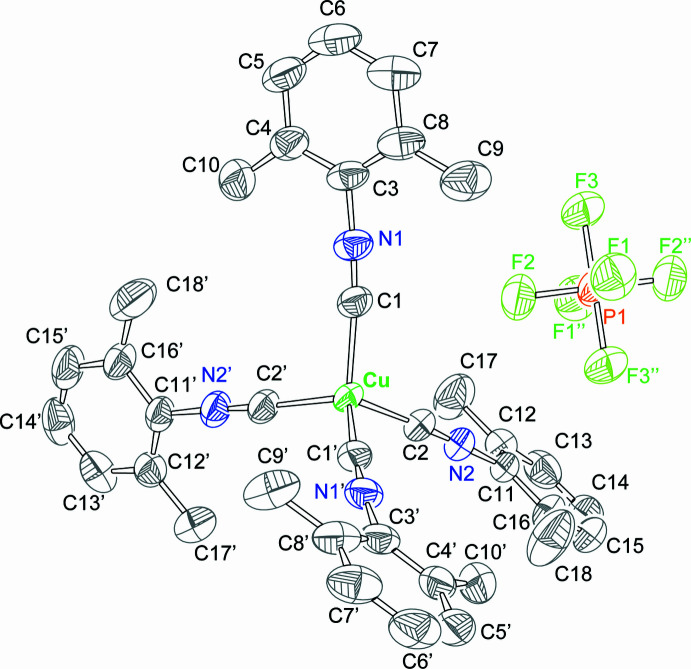
The structure of [Cu(CNX­yl)]_4_(PF_6_) with 50% probability ellipsoids. [Symmetry codes: (′) −*x* + 1, *y*, −*z* + 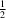; (′′) −*x* + 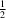, −*y* + 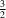, −*z* + 1.]

The crystals of [Cu(CNX­yl)_4_]PF_6_ were obtained by vapour diffusion using CH_2_Cl_2_/ether solvent mixture at 238 K. The compound crystallizes in the *C*2/*c* space group. The structure is presented in Fig. 1 ▸ and selected bond distances and angles are presented in Table 1 ▸. The mol­ecule occupies a special position (twofold rotation), with only half of the complex (and anion positioned on an inversion center) constituting an asymmetric unit. The metal center exhibits a slightly distorted tetra­hedral geometry, with C—Cu—C angles ranging between 105.02 (8) and 112.99 (11)°. Cu—C bonds of 1.9605 (18) and 1.9610 (18) Å are significantly longer than the Cu—C bonds in the previously described trigonal complex [Cu(CNX­yl)_3_]^+^ [1.908 (2)–1.919 (1) Å; Ferraro *et al.*, 2021 ▸]. Inter­estingly, pairs of isocyanides within the complex (C1N1Xyl and C2′N2′Xyl, C1′N1′Xyl and C2N2Xyl) exhibit coplanar arrangements of their aromatic rings. The angles between planes of coplanar pairs of isocyanide planes are ∼7°. This phenomenon is likely due to supra­molecular inter­actions (*vide infra*). A similar planarity was observed for the trigonal-planar [Cu(CNX­yl)_3_]^+^ (Ferraro *et al.*, 2021 ▸). All metrics associated with isocyanide ligands (C≡N bonds, CNC angles) are short and unexceptional.

## 3. Supra­molecular features

**Figure 2:**
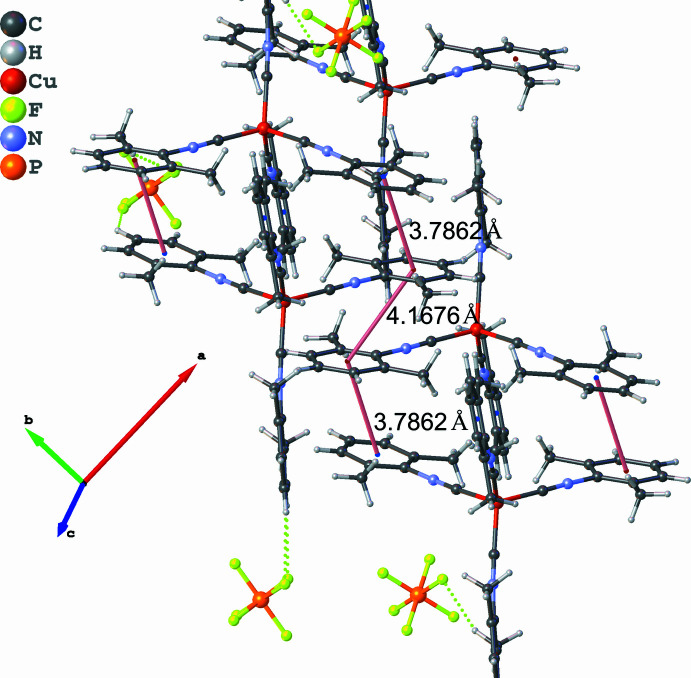
Supra­molecular structure of [Cu(CNX­yl)_4_]PF_6_ exhibiting π–π inter­actions (pink lines) and H⋯F inter­actions (yellow dashed lines).

The supra­molecular structure of [Cu(CNX­yl)_4_]PF_6_ (within one unit cell) is shown in Fig. 2 ▸. The drawing demonstrates a significant inter­molecular inter­action between neighboring complex mol­ecules. The inter­action involves offset π–π stacking, with centroid–centroid distance of 3.7862 (13) and 4.1676 (18) Å, with the latter distance being longer than expected (Janiak, 2000 ▸). Each mol­ecule is engaged in three such inter­actions, forming two perpendicular chains. This inter­action is likely responsible for the coplanar arrangement of two xylyl isocyanides in each complex, as it allows tighter packing. In addition, the structure demonstrates CH_3_(xyl­yl)⋯PF_6_ and CH*sp*^2^(xyl­yl)⋯PF_6_ inter­actions (∼2.4–2.6 Å).

## 4. Hirshfeld surface analysis

**Figure 3:**
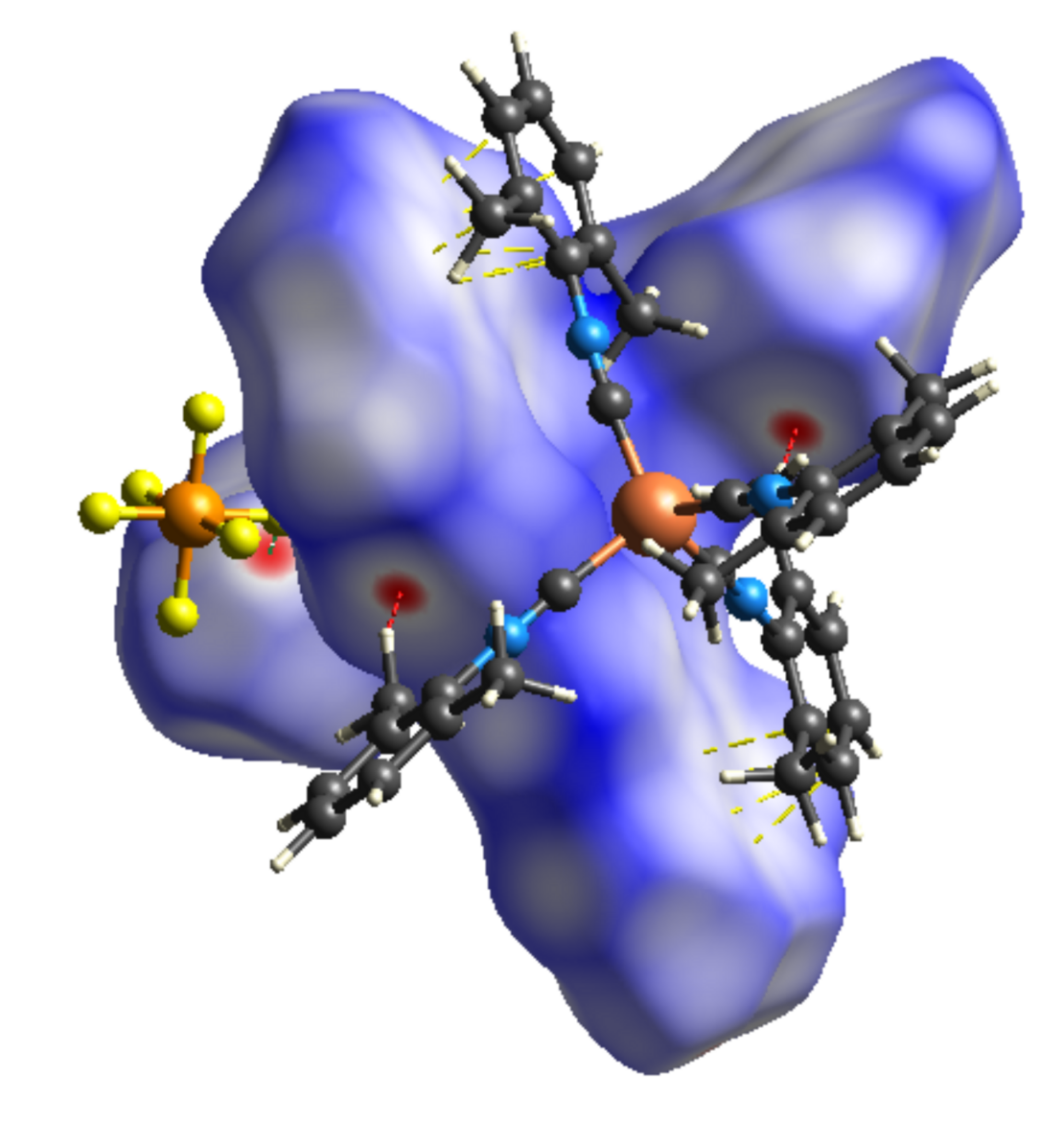
Hirshfeld surface of [Cu(CNX­yl)_4_]PF_6_ mapped with *d*_norm_. Close contacts shown are H⋯H (red), F⋯H/H⋯F (green), and C⋯C (yellow).

To qu­antify inter­molecular inter­actions influencing the packing of [Cu(CNX­yl)_4_]PF_6_, a Hirshfeld surface analysis was undertaken (Spackman & Jayatilaka, 2009 ▸) and the corresponding two-dimensional fingerprint plots (Spackman & McKinnon, 2002 ▸) were generated using *CrystalExplorer21.5* (Spackman *et al.*, 2021 ▸). Contacts are revealed by examining the distances from the Hirshfeld surface to the nearest atom inside the surface (*d*_i_) and outside the surface (*d*_e_). The *d*_norm_ map is the normalized contact distance using *d*_i_ and *d*_e_ normalized to the van der Waals radius. The *d*_norm_ map reveals contact regions that are closer than the van der Waals radii (red) to those that are longer (blue), where white is at the van der Waals radii. Fig. 3 ▸ shows H⋯H (red), F⋯H/H⋯F (green), and C⋯C (yellow) close contacts in [Cu(CNX­yl)_4_]PF_6_.

**Figure 4:**
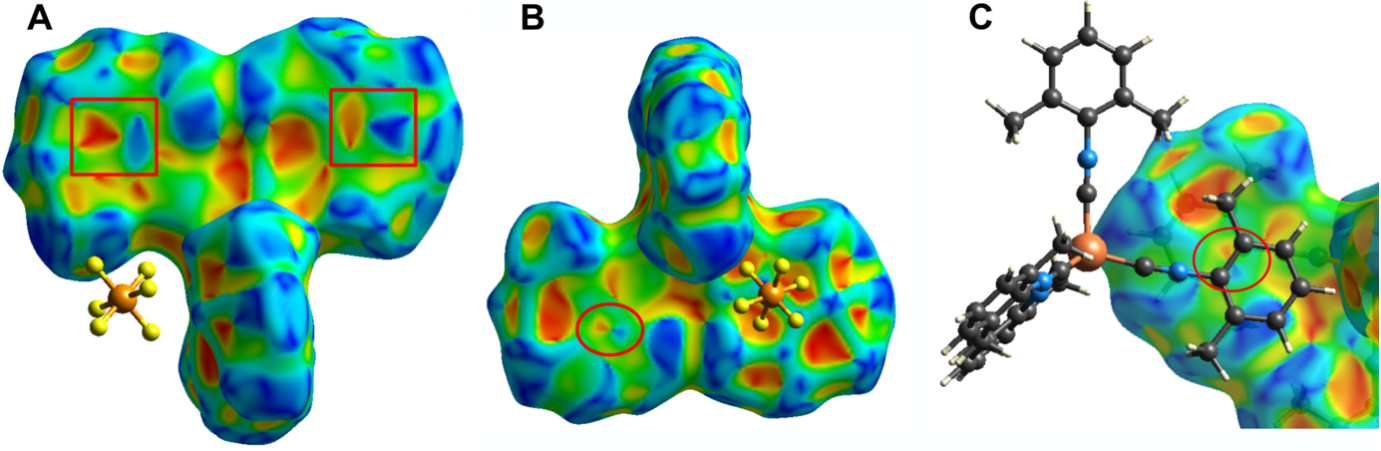
Hirshfeld surface mapped with shape-index. (*a*) The red squares highlight regions of large red and blue triangles over two xylyl groups (parallel to the paper). (*b*) After a 90° rotation of (*a*), there is a small red–blue triangle pair (red circle) suggesting a π–π stacking inter­action. The other xylyl ring has a neighboring PF_6_ ion, thus no π–π stacking inter­actions. (*c*) A zoomed in view of the red circle in (*b*), which shows the location of the small red–blue triangle pair aligns with the C—C bond of the two inter­acting xylyl rings.

The shape-index surface is useful for detecting π–π inter­actions, which are indicated as touching red–blue triangles (McKinnon *et al.*, 2004 ▸; Spackman & Jayatilaka, 2009 ▸). Fig. 4 ▸*a* shows a large red triangle and a large blue triangle over two xylyl rings (highlighted by red squares); however, the touching pattern appears less like triangles, but the alternating pattern and the 3.7862 Å centroid–centroid distance still indicates π–π stacking inter­actions (McKinnon *et al.*, 2004 ▸). Rotating the mol­ecule by 90°, one of the xylyl rings shows a small red–blue triangle pair directly above and below the bond between two carbon atoms (Fig. 4 ▸*b* and 4*c*). The fourth xylyl ring in Fig. 4 ▸*b* does not indicate π–π inter­actions because a PF_6_ ion is positioned above this ring. The xylyl rings in the red squares in Fig. 4 ▸*a* have a distance to the neighboring ring of 3.79 Å, which is a typical centroid–centroid distance (Janiak, 2000 ▸). The centroid–centroid distance shown in Fig. 4 ▸*c* is 4.1676 Å. Despite the unusually large centroid–centroid distance between these two xylyl rings, the shape-index surface suggests there is a π–π stacking inter­action.

**Figure 5:**
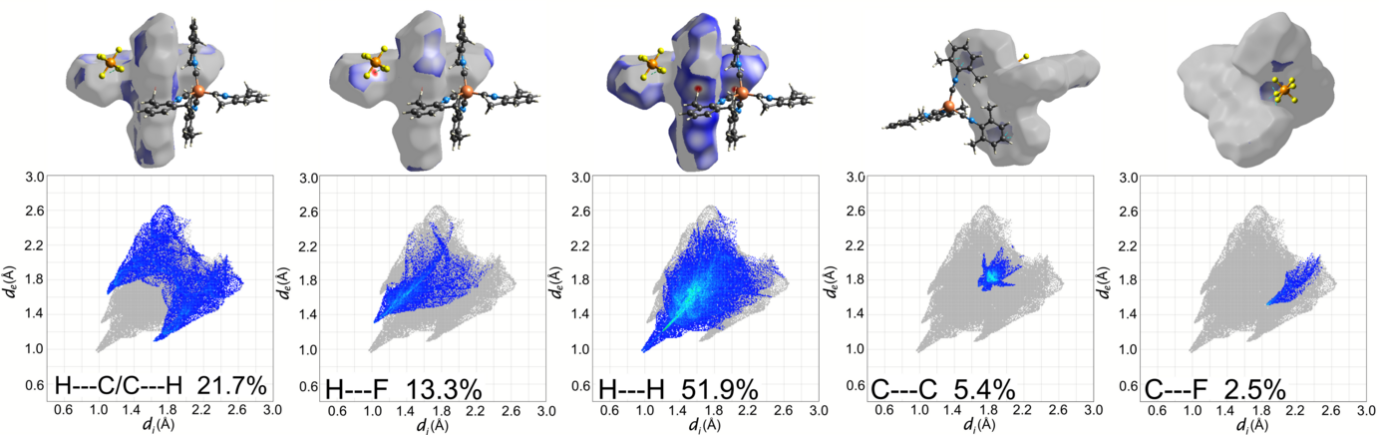
Fingerprint plots of [Cu(CNX­yl)_4_]PF_6_ with the corresponding *d*_map_ inter­actions above the plots.

We can qu­antify each inter­molecular inter­action type from the two-dimensional fingerprint plots (Fig. 5 ▸), which summarize the frequency of every combination of *d*_e_ and *d*_i_ pairs, while providing specific inter­actions and the relative area of the inter­actions (McKinnon *et al.*, 2004 ▸). The majority of the short contacts are H⋯H (51.9%), C⋯H/H⋯C (21.7%), and F⋯H/H⋯F (13.3%), which are the intense red areas on the *d*_map_ (Fig. 3 ▸). The C⋯C inter­actions (π–π stacking) for [Cu(CNX­yl)_4_] contribute 5.4%. For comparison, the tris complex, [Cu(CNX­yl)_3_](BF_4_) was also evaluated (CCDC #2073393; Ferraro *et al.*, 2021 ▸). The value of the C⋯C inter­action was 7.8%. The smaller percentage for [Cu(CNX­yl)_4_] may be due to the longer π–π inter­action (4.166 Å), while all of the π–π planar inter­actions in [Cu(CNX­yl)_3_] are ∼3.7 Å.

**Figure 6:**
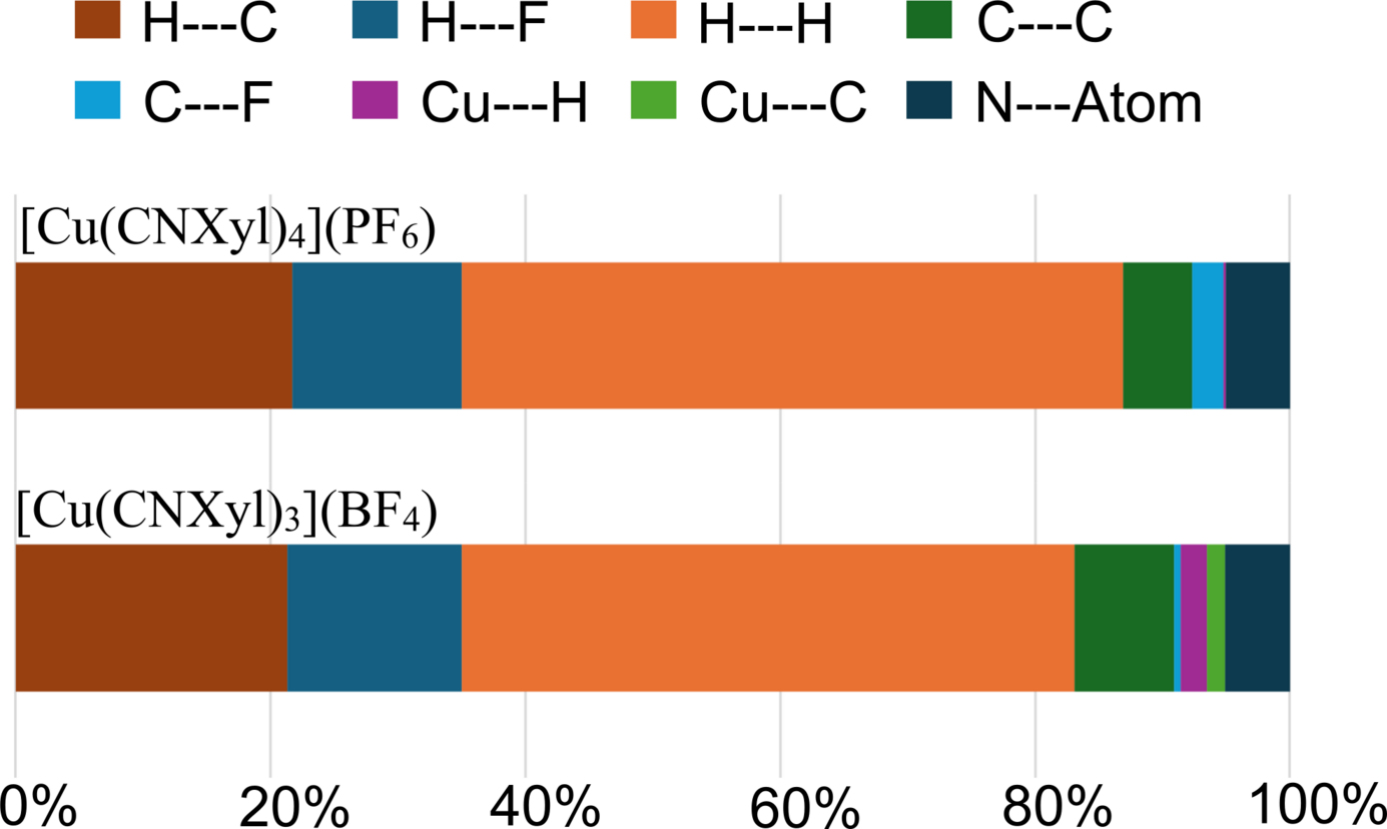
Percent contributions to the Hirshfeld surface area for various close inter­molecular contacts for complexes [Cu(CNX­yl)_4_]PF_6_ (this study) and [Cu(CNX­yl)_3_](BF_4_).

A summary of the inter­molecular inter­actions between the tris and tetra complexes are presented in Fig. 6 ▸. In addition to the difference in C⋯C inter­actions, the other significant differences include the addition of Cu⋯H/H⋯Cu and Cu⋯C/C⋯Cu inter­actions for [Cu(CNX­yl)_3_]. The trigonal-planer geometry of the tris complex provides access to the Cu atom. The other difference is the larger C⋯F/F⋯C inter­actions for [Cu(CNX­yl)_4_]PF_6_ at 2.5%, while only 0.5% for [Cu(CNX­yl)_3_](BF_4_). The additional inter­action with the counter-ion for [Cu(CNX­yl)_4_](PF_6_) is likely due to the reduced C⋯C inter­actions.

## 5. Database survey

Looking for related structures, we conducted two database searches in the Cambridge Structural Database (WebCSD, September 2025; Groom *et al.*, 2016 ▸). The first search focused on Cu^I^ complexes with xylyl isocyanide. While no tetra-coordinate [Cu(CNX­yl)_4_]^+^ were found, several tri-coordinate [Cu(CNX­yl)_3_]^+^ were observed, as described above. We have also searched for tetra-coordinate Cu^I^ complexes with phenyl isocyanide; this search revealed six tetra­hedral [Cu(CNAr)_4_]^+^ structures, in which CNAr was a *para*-substituted aryl isocyanide.

## 6. Synthesis and crystallization

[Cu(NCMe)_4_]PF_6_ (10 mg, 0.027 mmol, 1.0 equiv) was dissolved in aceto­nitrile (2 mL) and CNXyl [CNXyl = CN(2,6-MeC_6_H_3_)] (35.2 mg, 0.270 mmol, 10.0 equiv) was dissolved in aceto­nitrile (2 mL). Both solutions were cooled to −33 °C (238 K). The colorless solution of CNXyl was then added dropwise to a stirred colorless solution of [Cu(NCMe)_4_]PF_6_ producing a colourless solution. The reaction mixture was stirred for 1 h, after which volatiles were removed *in vacuo.* The product was obtained as a white solid (36.8 mg, 0.0502 mmol, 81%). This solid was recrystallized *via* vapor diffusion of ether into di­chloro­methane at 238 K to obtain colorless crystal suitable for X-ray crystallography. ^1^H NMR (298K, 400 MHz, CD_2_Cl_2_) δ 7.34 (*t*, *J* = 8 Hz, 1H), 7.22 (*d*, *J* = 8 Hz, 2H), 2.49 (*s*, 6H). ^13^C NMR (101 MHz, CD_2_Cl_2_) δ 146.47 (*C*NX­yl), 136.56, 131.11, 129.01, 126.09, 19.24. IR (cm^−1^, selected peaks) 2153 (*vs*, C≡NX­yl).

## 7. Refinement

Crystal data, data collection and structure refinement details are summarized in Table 2 ▸. The hydrogen atoms were positioned with idealized geometry and refined isotropically using a riding model.

## Supplementary Material

Crystal structure: contains datablock(s) I. DOI: 10.1107/S2056989025009867/oo2014sup1.cif

Structure factors: contains datablock(s) I. DOI: 10.1107/S2056989025009867/oo2014Isup2.hkl

CCDC reference: 2500684

Additional supporting information:  crystallographic information; 3D view; checkCIF report

## Figures and Tables

**Table 1:** Selected geometric parameters (Å, °)

Cu1—C1	1.9605 (18)	N1—C1	1.152 (2)
Cu1—C2	1.9610 (18)	N2—C2	1.154 (2)
			
C1—Cu1—C1^i^	112.99 (11)	C1—N1—C3	176.48 (18)
C1—Cu1—C2	111.56 (7)	C2—N2—C11	177.1 (2)
C1—Cu1—C2^i^	105.02 (8)	N1—C1—Cu1	174.35 (17)
C2^i^—Cu1—C2	110.85 (11)	N2—C2—Cu1	176.58 (16)

Symmetry code: (i) 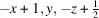.

**Table 2:** Experimental details

Crystal data	
Chemical formula	[Cu(C_9_H_9_N)_4_]PF_6_
*M*_r_	733.20
Crystal system, space group	Monoclinic, *C*2/*c*
Temperature (K)	100
*a*, *b*, *c* (Å)	25.4731 (7), 10.7452 (3), 15.1475 (4)
β (°)	121.770 (1)
*V* (Å^3^)	3524.86 (17)
*Z*	4
Radiation type	Mo *K*α
μ (mm^−1^)	0.73
Crystal size (mm)	0.20 × 0.15 × 0.10

Data collection	
Diffractometer	Bruker D8 VENTURE
Absorption correction	Multi-scan (*SADABS*; Krause *et al.*, 2015 ▸)
*T*_min_, *T*_max_	0.724, 0.742
No. of measured, independent and observed [*I* > 2σ(*I*)] reflections	92973, 4046, 3500
*R*_int_	0.052
(sin θ/λ)_max_ (Å^−1^)	0.650

Refinement	
*R*[*F*^2^ > 2σ(*F*^2^)], *wR*(*F*^2^), *S*	0.032, 0.101, 1.07
No. of reflections	4046
No. of parameters	223
H-atom treatment	H-atom parameters constrained
Δρ_max_, Δρ_min_ (e Å^−3^)	0.24, −0.17

Computer programs: *APEX6* and *SAINT* (Bruker, 2024 ▸), *SHELXT* (Sheldrick, 2015*a* ▸), *SHELXL2019/3* (Sheldrick, 2015*b* ▸) and *OLEX2* (Dolomanov *et al.*, 2009 ▸).

## supplementary crystallographic information

### Tetrakis(2,6-dimethylphenylisocyanide)copper(I) hexafluorophosphate Crystal data

**Table:**

[Cu(C_9_H_9_N)_4_]PF_6_	*F*(000) = 1512
*M**_r_* = 733.20	*D*_x_ = 1.382 Mg m^−^^3^
Monoclinic, *C*2/*c*	Mo *K*α radiation, λ = 0.71073 Å
*a* = 25.4731 (7) Å	Cell parameters from 9948 reflections
*b* = 10.7452 (3) Å	θ = 2.3–27.0°
*c* = 15.1475 (4) Å	µ = 0.73 mm^−^^1^
β = 121.770 (1)°	*T* = 100 K
*V* = 3524.86 (17) Å^3^	Prism, colourless
*Z* = 4	0.2 × 0.15 × 0.1 mm

### Tetrakis(2,6-dimethylphenylisocyanide)copper(I) hexafluorophosphate Data collection

**Table:**

Bruker D8 VENTURE diffractometer	4046 independent reflections
Radiation source: microfocus sealed tube, Incoatec IµS	3500 reflections with *I* > 2σ(*I*)
Multilayer mirror monochromator	*R*_int_ = 0.052
φ and ω scans	θ_max_ = 27.5°, θ_min_ = 2.1°
Absorption correction: multi-scan (SADABS; Krause *et al.*, 2015)	*h* = −33→33
*T*_min_ = 0.724, *T*_max_ = 0.742	*k* = −13→13
92973 measured reflections	*l* = −19→19

### Tetrakis(2,6-dimethylphenylisocyanide)copper(I) hexafluorophosphate Refinement

**Table:**

Refinement on *F*^2^	0 restraints
Least-squares matrix: full	Hydrogen site location: inferred from neighbouring sites
*R*[*F*^2^ > 2σ(*F*^2^)] = 0.032	H-atom parameters constrained
*wR*(*F*^2^) = 0.101	*w* = 1/[σ^2^(*F*_o_^2^) + (0.0527*P*)^2^ + 1.8864*P*] where *P* = (*F*_o_^2^ + 2*F*_c_^2^)/3
*S* = 1.07	(Δ/σ)_max_ = 0.001
4046 reflections	Δρ_max_ = 0.24 e Å^−^^3^
223 parameters	Δρ_min_ = −0.17 e Å^−^^3^

### Tetrakis(2,6-dimethylphenylisocyanide)copper(I) hexafluorophosphate Special details

**Table:**

Experimental. A suitable crystal was mounted on a Bruker D8 Venture diffractometer with kappa geometry, an Incoatec IµS micro-focus source X-ray tube (Mo K_α_ radiation), and a multilayer mirror for monochromatization. The diffraction intensities were measured using a Photon III CPAD area detector. Data were acquired at 100 K with an Oxford 800 Cryostream low-temperature apparatus. Using APEX6 v2024.9-0, the intensities were integrated using SAINT V8.40b and a multiscan absorption correction was applied with SADABS-2016/2. The crystal structure was solved and refined using SHELXT (Sheldrick, 2015a) and least-squares refinement with SHELXL-2019/3 (Sheldrick, 2015b) running under Olex2 (Dolomanov *et al.*, 2009). All non–hydrogen atoms were refined anisotropically.
Geometry. All esds (except the esd in the dihedral angle between two l.s. planes) are estimated using the full covariance matrix. The cell esds are taken into account individually in the estimation of esds in distances, angles and torsion angles; correlations between esds in cell parameters are only used when they are defined by crystal symmetry. An approximate (isotropic) treatment of cell esds is used for estimating esds involving l.s. planes.
Refinement. The 200 and 111 reflections were omitted due to being blocked or partially blocked by the beam stop. The other three reflections were omitted for large Fcalc. Non-merohedral and pseudo-merohedral twinning was looked for but did not find a suitable twin law or a second domain.

### Tetrakis(2,6-dimethylphenylisocyanide)copper(I) hexafluorophosphate Fractional atomic coordinates and isotropic or equivalent isotropic displacement parameters (Å^2^)

**Table:**

	*x*	*y*	*z*	*U*_iso_*/*U*_eq_	
Cu1	0.500000	0.33279 (3)	0.250000	0.04408 (11)	
P1	0.250000	0.750000	0.500000	0.0623 (2)	
F1	0.24423 (7)	0.60646 (15)	0.51955 (11)	0.0860 (4)	
F2	0.18029 (6)	0.74986 (16)	0.40429 (10)	0.0844 (4)	
F3	0.27340 (7)	0.71339 (17)	0.42396 (10)	0.0837 (4)	
N1	0.44524 (7)	0.17581 (14)	0.35511 (12)	0.0524 (4)	
N2	0.40387 (7)	0.49851 (15)	0.06716 (11)	0.0532 (4)	
C1	0.46296 (8)	0.23207 (18)	0.31124 (13)	0.0507 (4)	
C2	0.43813 (8)	0.43636 (19)	0.13574 (13)	0.0527 (4)	
C3	0.42734 (9)	0.10442 (18)	0.41253 (15)	0.0532 (4)	
C4	0.45639 (9)	0.1277 (2)	0.51896 (16)	0.0615 (5)	
C5	0.43930 (11)	0.0512 (3)	0.57417 (18)	0.0756 (7)	
H5	0.461572	0.064477	0.658697	0.091*	
C6	0.39537 (12)	−0.0403 (2)	0.5258 (2)	0.0799 (7)	
H6	0.383238	−0.099785	0.571846	0.096*	
C7	0.36633 (12)	−0.0582 (2)	0.4197 (2)	0.0742 (6)	
H7	0.330330	−0.130185	0.381418	0.089*	
C8	0.38227 (11)	0.01436 (19)	0.36079 (17)	0.0618 (5)	
C9	0.35095 (14)	−0.0037 (3)	0.2452 (2)	0.0835 (7)	
H9A	0.384325	−0.040329	0.226918	0.125*	
H9B	0.333033	0.085889	0.205852	0.125*	
H9C	0.312661	−0.069704	0.218740	0.125*	
C10	0.50276 (10)	0.2294 (3)	0.56955 (18)	0.0797 (7)	
H10A	0.538384	0.211912	0.563170	0.119*	
H10B	0.516293	0.234284	0.643171	0.119*	
H10C	0.484074	0.308693	0.535562	0.119*	
C11	0.36347 (8)	0.57934 (18)	−0.01298 (12)	0.0493 (4)	
C12	0.33917 (9)	0.6776 (2)	0.01145 (16)	0.0580 (5)	
C13	0.30131 (10)	0.7600 (2)	−0.0679 (2)	0.0714 (6)	
H13	0.281228	0.840924	−0.051329	0.086*	
C14	0.28884 (10)	0.7408 (2)	−0.16689 (19)	0.0748 (7)	
H14	0.258547	0.806384	−0.229230	0.090*	
C15	0.31333 (11)	0.6422 (3)	−0.18888 (16)	0.0768 (7)	
H15	0.302400	0.628829	−0.269041	0.092*	
C16	0.35194 (10)	0.5577 (2)	−0.11239 (14)	0.0643 (5)	
C17	0.35335 (15)	0.6937 (3)	0.1207 (2)	0.0968 (9)	
H17A	0.342084	0.617649	0.142587	0.145*	
H17B	0.329636	0.763969	0.123090	0.145*	
H17C	0.397576	0.709823	0.167384	0.145*	
C18	0.38040 (17)	0.4495 (4)	−0.1342 (2)	0.1130 (12)	
H18A	0.367041	0.362761	−0.112933	0.169*	
H18B	0.430846	0.458695	−0.088595	0.169*	
H18C	0.364182	0.447586	−0.217197	0.169*	

### Tetrakis(2,6-dimethylphenylisocyanide)copper(I) hexafluorophosphate Atomic displacement parameters (Å^2^)

**Table:**

	*U*^11^	*U*^22^	*U*^33^	*U*^12^	*U*^13^	*U*^23^
Cu1	0.04500 (17)	0.0608 (2)	0.02891 (14)	0.000	0.02116 (12)	0.000
P1	0.0586 (4)	0.0893 (6)	0.0406 (3)	0.0024 (4)	0.0273 (3)	0.0124 (3)
F1	0.0929 (10)	0.0894 (10)	0.0810 (9)	0.0009 (8)	0.0494 (8)	0.0180 (8)
F2	0.0618 (7)	0.1242 (12)	0.0539 (7)	0.0025 (7)	0.0212 (6)	0.0119 (7)
F3	0.0869 (9)	0.1199 (11)	0.0611 (7)	0.0040 (8)	0.0505 (7)	0.0068 (8)
N1	0.0543 (8)	0.0602 (9)	0.0554 (9)	0.0128 (7)	0.0376 (7)	0.0126 (7)
N2	0.0500 (8)	0.0657 (9)	0.0377 (7)	−0.0025 (7)	0.0189 (6)	0.0055 (7)
C1	0.0496 (9)	0.0642 (11)	0.0448 (9)	0.0087 (8)	0.0294 (8)	0.0075 (8)
C2	0.0517 (9)	0.0687 (11)	0.0362 (8)	−0.0021 (8)	0.0222 (7)	0.0021 (8)
C3	0.0597 (10)	0.0593 (10)	0.0600 (10)	0.0180 (8)	0.0449 (9)	0.0184 (8)
C4	0.0512 (10)	0.0862 (14)	0.0564 (10)	0.0201 (10)	0.0348 (9)	0.0187 (10)
C5	0.0691 (13)	0.1117 (19)	0.0617 (12)	0.0289 (13)	0.0453 (11)	0.0335 (13)
C6	0.0910 (16)	0.0878 (16)	0.0933 (17)	0.0238 (14)	0.0709 (15)	0.0371 (14)
C7	0.0938 (16)	0.0618 (12)	0.1006 (18)	0.0089 (11)	0.0742 (15)	0.0136 (12)
C8	0.0822 (13)	0.0541 (10)	0.0757 (13)	0.0104 (9)	0.0598 (12)	0.0070 (9)
C9	0.115 (2)	0.0801 (15)	0.0827 (16)	−0.0166 (14)	0.0705 (16)	−0.0189 (13)
C10	0.0522 (11)	0.122 (2)	0.0613 (13)	0.0046 (12)	0.0275 (10)	0.0032 (13)
C11	0.0442 (8)	0.0626 (10)	0.0353 (7)	−0.0056 (7)	0.0169 (7)	0.0067 (7)
C12	0.0475 (9)	0.0738 (13)	0.0513 (10)	−0.0034 (8)	0.0250 (8)	0.0017 (9)
C13	0.0499 (10)	0.0734 (14)	0.0846 (16)	0.0029 (9)	0.0310 (11)	0.0117 (11)
C14	0.0505 (11)	0.0899 (16)	0.0669 (13)	−0.0002 (11)	0.0191 (10)	0.0337 (12)
C15	0.0661 (13)	0.1111 (19)	0.0409 (10)	−0.0056 (13)	0.0198 (9)	0.0199 (11)
C16	0.0625 (11)	0.0851 (14)	0.0382 (9)	−0.0007 (10)	0.0216 (8)	0.0016 (9)
C17	0.0942 (19)	0.136 (3)	0.0633 (15)	0.0190 (17)	0.0438 (14)	−0.0132 (15)
C18	0.136 (3)	0.134 (3)	0.0610 (15)	0.033 (2)	0.0463 (17)	−0.0132 (16)

### Tetrakis(2,6-dimethylphenylisocyanide)copper(I) hexafluorophosphate Geometric parameters (Å, º)

**Table:**

Cu1—C1	1.9605 (18)	C8—C9	1.506 (3)
Cu1—C1^i^	1.9606 (18)	C9—H9A	1.0970
Cu1—C2	1.9610 (18)	C9—H9B	1.0970
Cu1—C2^i^	1.9610 (18)	C9—H9C	1.0970
P1—F1^ii^	1.5919 (15)	C10—H10A	0.9800
P1—F1	1.5919 (15)	C10—H10B	0.9800
P1—F2	1.5950 (13)	C10—H10C	0.9800
P1—F2^ii^	1.5951 (13)	C11—C12	1.370 (3)
P1—F3^ii^	1.5998 (13)	C11—C16	1.393 (3)
P1—F3	1.5999 (13)	C12—C13	1.392 (3)
N1—C1	1.152 (2)	C12—C17	1.506 (3)
N1—C3	1.404 (2)	C13—H13	1.1030
N2—C2	1.154 (2)	C13—C14	1.375 (4)
N2—C11	1.404 (2)	C14—H14	1.1030
C3—C4	1.397 (3)	C14—C15	1.357 (4)
C3—C8	1.386 (3)	C15—H15	1.1030
C4—C5	1.395 (3)	C15—C16	1.391 (3)
C4—C10	1.491 (4)	C16—C18	1.497 (4)
C5—H5	1.1030	C17—H17A	0.9800
C5—C6	1.376 (4)	C17—H17B	0.9800
C6—H6	1.1030	C17—H17C	0.9800
C6—C7	1.384 (4)	C18—H18A	1.0970
C7—H7	1.1030	C18—H18B	1.0970
C7—C8	1.397 (3)	C18—H18C	1.0970
			
C1—Cu1—C1^i^	112.99 (11)	C8—C9—H9A	109.5
C1^i^—Cu1—C2^i^	111.56 (7)	C8—C9—H9B	109.5
C1—Cu1—C2	111.56 (7)	C8—C9—H9C	109.5
C1—Cu1—C2^i^	105.02 (8)	H9A—C9—H9B	109.5
C1^i^—Cu1—C2	105.01 (8)	H9A—C9—H9C	109.5
C2^i^—Cu1—C2	110.85 (11)	H9B—C9—H9C	109.5
F1—P1—F1^ii^	180.0	C4—C10—H10A	109.5
F1^ii^—P1—F2^ii^	90.03 (8)	C4—C10—H10B	109.5
F1^ii^—P1—F2	89.97 (8)	C4—C10—H10C	109.5
F1—P1—F2	90.03 (8)	H10A—C10—H10B	109.5
F1—P1—F2^ii^	89.97 (8)	H10A—C10—H10C	109.5
F1—P1—F3^ii^	89.91 (8)	H10B—C10—H10C	109.5
F1^ii^—P1—F3	89.90 (8)	C12—C11—N2	118.23 (16)
F1—P1—F3	90.09 (8)	C12—C11—C16	123.53 (18)
F1^ii^—P1—F3^ii^	90.10 (8)	C16—C11—N2	118.21 (18)
F2—P1—F2^ii^	180.0	C11—C12—C13	117.58 (19)
F2^ii^—P1—F3	89.78 (8)	C11—C12—C17	120.2 (2)
F2—P1—F3	90.22 (8)	C13—C12—C17	122.2 (2)
F2—P1—F3^ii^	89.78 (8)	C12—C13—H13	119.9
F2^ii^—P1—F3^ii^	90.22 (8)	C14—C13—C12	120.2 (2)
F3^ii^—P1—F3	180.0	C14—C13—H13	119.9
C1—N1—C3	176.48 (18)	C13—C14—H14	119.6
C2—N2—C11	177.1 (2)	C15—C14—C13	120.8 (2)
N1—C1—Cu1	174.35 (17)	C15—C14—H14	119.6
N2—C2—Cu1	176.58 (16)	C14—C15—H15	119.3
C4—C3—N1	118.02 (19)	C14—C15—C16	121.3 (2)
C8—C3—N1	118.27 (17)	C16—C15—H15	119.3
C8—C3—C4	123.71 (18)	C11—C16—C18	121.2 (2)
C3—C4—C10	121.26 (19)	C15—C16—C11	116.5 (2)
C5—C4—C3	116.3 (2)	C15—C16—C18	122.4 (2)
C5—C4—C10	122.4 (2)	C12—C17—H17A	109.5
C4—C5—H5	119.2	C12—C17—H17B	109.5
C6—C5—C4	121.6 (2)	C12—C17—H17C	109.5
C6—C5—H5	119.2	H17A—C17—H17B	109.5
C5—C6—H6	119.8	H17A—C17—H17C	109.5
C5—C6—C7	120.3 (2)	H17B—C17—H17C	109.5
C7—C6—H6	119.8	C16—C18—H18A	109.5
C6—C7—H7	119.7	C16—C18—H18B	109.5
C6—C7—C8	120.5 (2)	C16—C18—H18C	109.5
C8—C7—H7	119.7	H18A—C18—H18B	109.5
C3—C8—C7	117.4 (2)	H18A—C18—H18C	109.5
C3—C8—C9	121.42 (19)	H18B—C18—H18C	109.5
C7—C8—C9	121.1 (2)		
			
N1—C3—C4—C5	−177.29 (17)	C6—C7—C8—C9	−179.8 (2)
N1—C3—C4—C10	3.0 (3)	C8—C3—C4—C5	2.2 (3)
N1—C3—C8—C7	178.19 (17)	C8—C3—C4—C10	−177.48 (19)
N1—C3—C8—C9	−2.6 (3)	C10—C4—C5—C6	178.5 (2)
N2—C11—C12—C13	177.32 (17)	C11—C12—C13—C14	0.7 (3)
N2—C11—C12—C17	−3.0 (3)	C12—C11—C16—C15	−0.2 (3)
N2—C11—C16—C15	−177.91 (18)	C12—C11—C16—C18	179.3 (3)
N2—C11—C16—C18	1.6 (3)	C12—C13—C14—C15	−0.4 (3)
C3—C4—C5—C6	−1.2 (3)	C13—C14—C15—C16	−0.3 (4)
C4—C3—C8—C7	−1.3 (3)	C14—C15—C16—C11	0.5 (3)
C4—C3—C8—C9	177.9 (2)	C14—C15—C16—C18	−178.9 (3)
C4—C5—C6—C7	−0.6 (4)	C16—C11—C12—C13	−0.4 (3)
C5—C6—C7—C8	1.6 (4)	C16—C11—C12—C17	179.3 (2)
C6—C7—C8—C3	−0.6 (3)	C17—C12—C13—C14	−179.0 (2)

Symmetry codes: (i) −*x*+1, *y*, −*z*+1/2; (ii) −*x*+1/2, −*y*+3/2, −*z*+1.
